# Correction: Psychological characteristics and the mediating role of the 5C Model in explaining students’ COVID-19 vaccination intention

**DOI:** 10.1371/journal.pone.0259922

**Published:** 2021-11-09

**Authors:** Annelot Wismans, Roy Thurik, Rui Baptista, Marcus Dejardin, Frank Janssen, Ingmar Franken

In Fig 1, the lines in the legend have been omitted. Please see the correct Fig 1 here.

**Fig 1:**
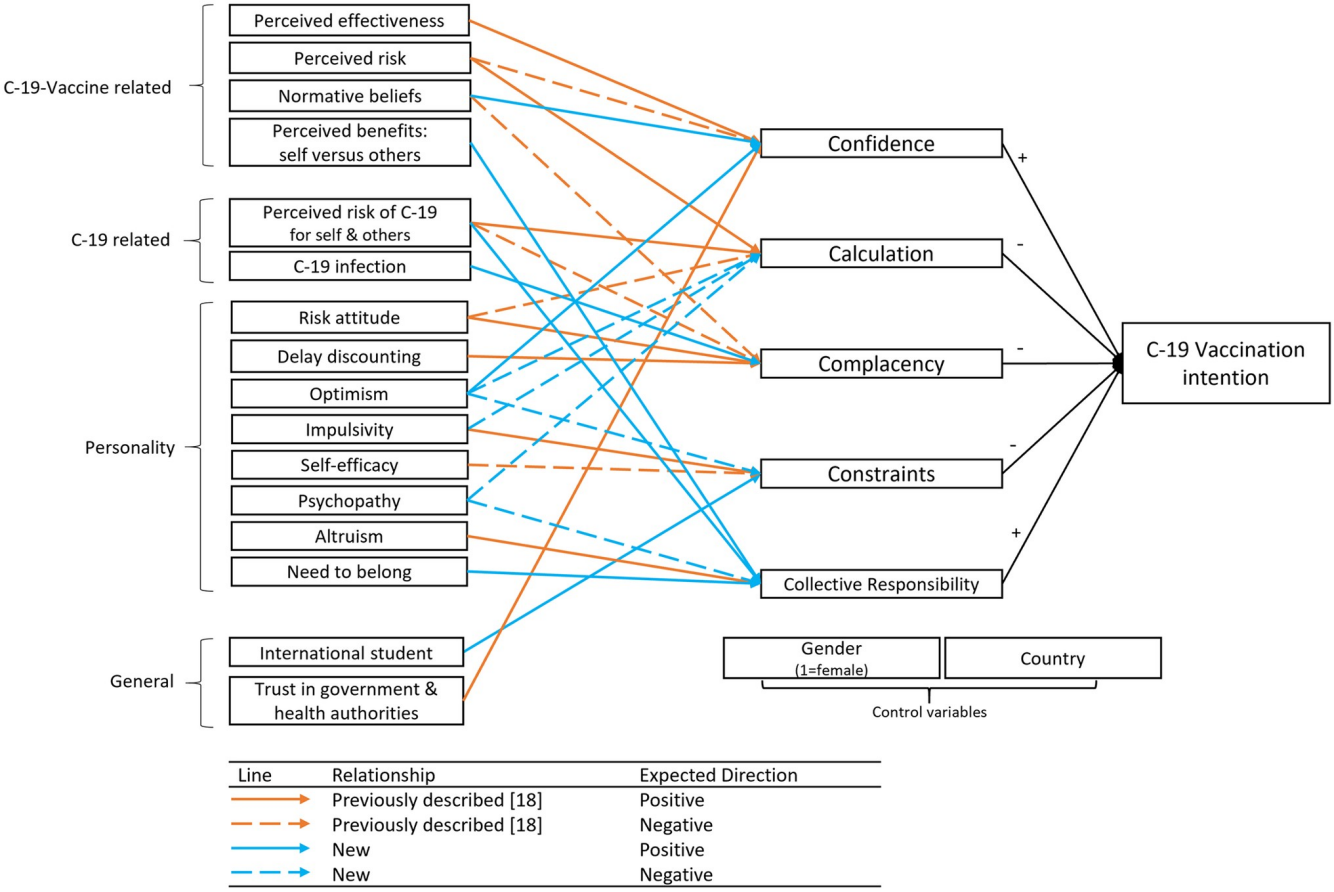
Overview of expected mediation relationships. Direct effects are excluded for clarity reasons. (C-19 = COVID-19).

The images for Figs 2 and 3 are incorrectly switched. The image that appears as Fig 2 should be Fig 3, and the image that appears as Fig 3 should be Fig 2. The figure captions appear in the correct order.

**Fig 2:**
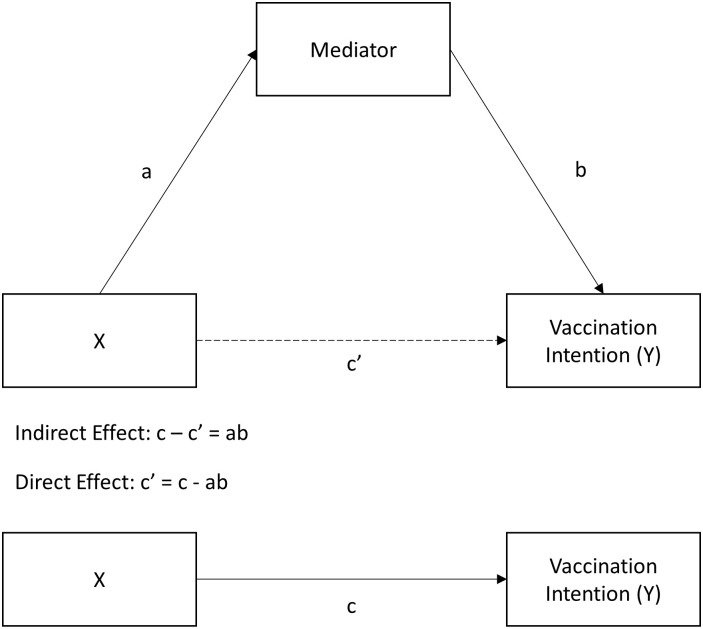
All paths involved in the mediation analyses, excluding covariates.

**Fig 3:**
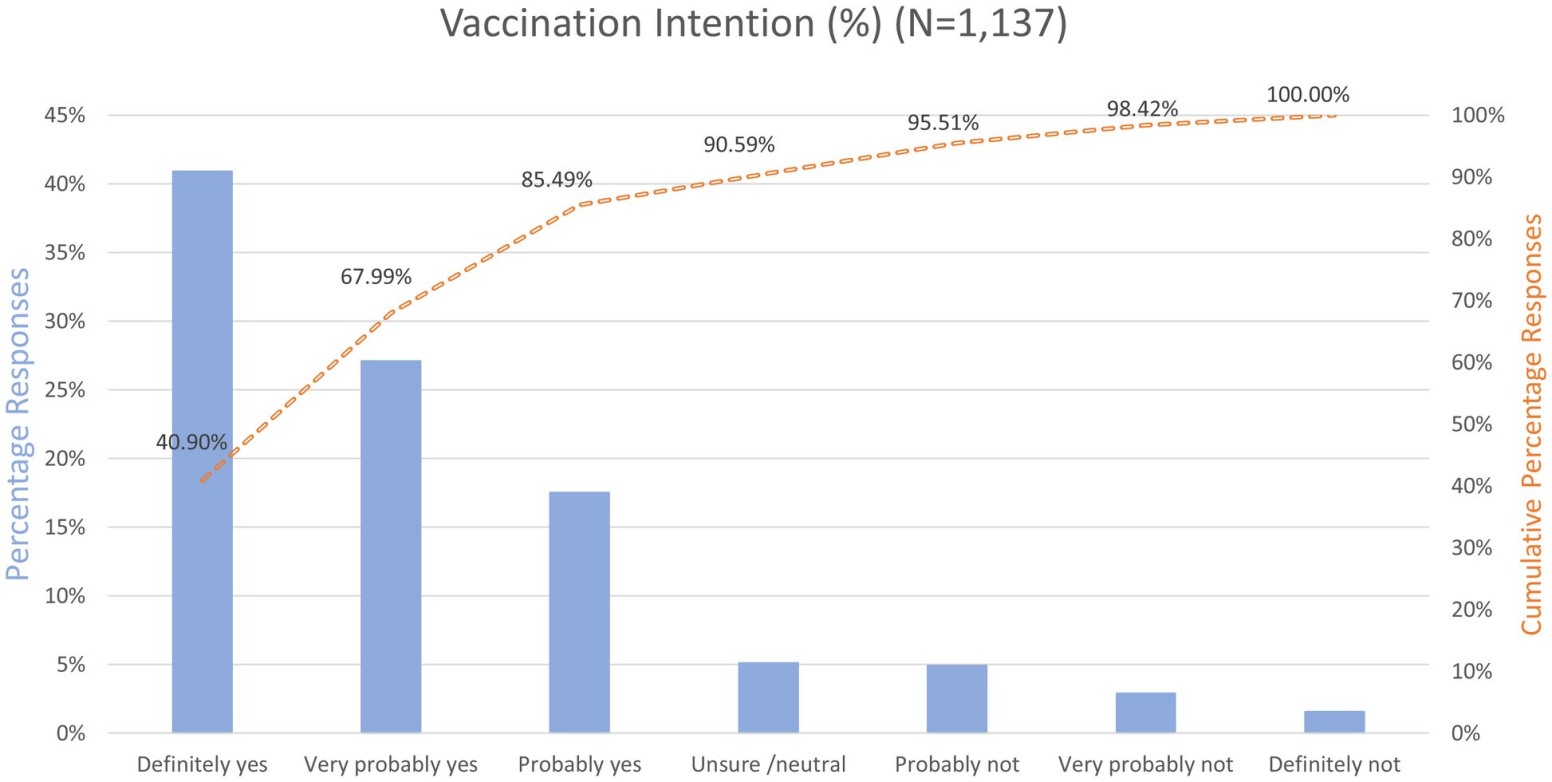
Vaccination intention in percentages per category and cumulative percentages.

## References

[1] Wismans A, Thurik R, Baptista R, Dejardin M, Janssen F, Franken I (2021) Psychological characteristics and the mediating role of the 5C Model in explaining students’ COVID-19 vaccination intention. PLoS ONE 16(8): e0255382. doi: 10.1371/journal.pone.0255382 34379648 PMC8357093

