# Supplementary material for: Psychological characteristics and the mediating role of the 5C Model in explaining students’ COVID-19 vaccination intention
Source: PLoS One. 2021 Aug 11;16(8):e0255382. doi: 10.1371/journal.pone.0255382 (PMC8357093; doi:10.1371/journal.pone.0255382)
Supplement: Attachment — Submitted filename: Comments for PLoS paper.docx [file pone.0255382.s001.docx]

**Comments**

The paper examines an interesting area, as it examines the psychological characteristics associated with students’ COVID-19 vaccination intention. Having read through the paper, I do not believe it needs any grammar editing.

Having read through the article, I have the following comments and suggestions.

**Overall**

The article has too many words in its current state. The authors should try to reduce the word count of the article.

**Title**

The title should reflect the 5C model used to make it catch the attention of the readers.

**Abstract**

As the study is a quantitative one, the abstract should contain the relevant coefficients showing the relationships between variables.

**Introduction**

A stronger justification should be given for using university students a representation of young persons, as they are only a fraction of all young persons.

Also, more information should be given about the 5C model. A brief summary of the theory should be added to the paper for readers not already familiar with the model.

**Methodology**

The methodology is adequate to answer the research objectives.

**Discussion and Conclusion**

This section is also adequate.
